# HTLV-1-induced leukotriene B4 secretion by T cells promotes T cell recruitment and virus propagation

**DOI:** 10.1038/ncomms15890

**Published:** 2017-06-22

**Authors:** Florent Percher, Céline Curis, Eléonore Pérès, Maria Artesi, Nicolas Rosewick, Patricia Jeannin, Antoine Gessain, Olivier Gout, Renaud Mahieux, Pierre-Emmanuel Ceccaldi, Anne Van den Broeke, Madeleine Duc Dodon, Philippe V. Afonso

**Affiliations:** 1 Unité d'Epidémiologie et Physiopathologie des Virus Oncogènes, Département de Virologie, Institut Pasteur, Paris F-75015, France; 2 Centre National de la Recherche Scientifique (CNRS) UMR 3569, Paris F-75015, France; 3 Université Paris Diderot, Sorbonne Paris Cité, Paris F-75013, France; 4 Laboratoire de Biologie et Modélisation de la Cellule, ENS de Lyon, INSERM U1210 CNRS-UCBL UMR 5239, UMS 3444 SFR Biosciences-Lyon, Lyon F-69007, France; 5 Unit of Animal Genomics, Groupe Interdisciplinaire Génoprotéomique Appliquée (GIGA), Université de Liège, Liège B-4000, Belgium; 6 Laboratory of Experimental Hematology, Institut Jules Bordet, Université Libre de Bruxelles, Brussels B-1000, Belgium; 7 Service de Neurologie, Fondation Ophtalmologique Adolphe de Rothschild, Paris F-75019, France; 8 Equipe Oncogenèse Rétrovirale, ENS de Lyon, and Equipe Labélisée Ligue Nationale Contre le Cancer, Centre International de Recherche en Infectiologie, INSERM U1111, CNRS UMR 5308, Lyon F-69007, France

## Abstract

The human T-lymphotropic virus type 1 (HTLV-1) is efficiently transmitted through cellular contacts. While the molecular mechanisms of viral cell-to-cell propagation have been extensively studied *in vitro*, those facilitating the encounter between infected and target cells remain unknown. In this study, we demonstrate that HTLV-1-infected CD4 T cells secrete a potent chemoattractant, leukotriene B4 (LTB4). LTB4 secretion is dependent on Tax-induced transactivation of the *pla2g4c* gene, which encodes the cytosolic phospholipase A2 gamma. Inhibition of LTB4 secretion or LTB4 receptor knockdown on target cells reduces T-cell recruitment, cellular contact formation and virus propagation *in vitro*. Finally, blocking the synthesis of LTB4 in a humanized mouse model of HTLV-1 infection significantly reduces proviral load. This results from a decrease in the number of infected clones while their expansion is not impaired. This study shows the critical role of LTB4 secretion in HTLV-1 transmission both *in vitro* and *in vivo*.

## Abstract (web summary)

HTLV-1 predominantly spreads through direct cell-cell contacts, but mechanisms of target cell recruitment are unclear. Here, the authors show that HTLV-1 infected T-cells secrete leukotriene B4, which recruits T-cells, facilitates HTLV-1 transmission *in vitro*, and increases the number of infected clones in mice.

The human T-lymphotropic virus type 1 (HTLV-1) is estimated to infect 5–20 million people worldwide^1^. Among HTLV-1-infected individuals, 90–95% remain asymptomatic throughout their lives. Nevertheless, HTLV-1 is the aetiological agent of many severe diseases, ranging from an aggressive lymphoproliferation, the adult T-cell leukaemia/lymphoma (ATL), to inflammatory syndromes, such as myositis, uveitis, infective dermatitis and a neurodegenerative disease called HTLV-1-associated myelopathy or tropical spastic paraparesis (HAM/TSP)^2^. HTLV-1-associated pathologies often develop in individuals with high proviral load (PVL), that is, elevated frequency of infected cells, mostly CD4 T cells^3^,^4^.

A major feature of HTLV-1 is that infection almost exclusively occurs through cell-associated virus. First, there is no viraemia associated with HTLV-1 infection, HTLV-1 genomic RNA is rarely detected in the plasma of infected individuals^5^. In addition, it is estimated that 10^6^ HTLV-1 particles are needed to infect one primary activated lymphocyte *in vitro*, underlining the low infectivity of the viral particles^6^. *In vivo*, contamination through blood transfusion requires the transfer of at least 10^8^ PBMCs^7^, hence the probability of HTLV-1 infection on leukoreduction is virtually null^8^,^9^. Dendritic cells seem to be the only cell type sensitive to infection through free viral particles^10^, although infection is still poorly efficient *in vitro*^11^.

Several non-exclusive mechanisms of viral transmission during cell–cell contact have been described: virus can spread through viral synapses, cellular conduits or transferred embedded in a viral biofilm^12^,^13^,^14^. The mechanisms facilitating the encounter between an infected cell and a target cell remain unclear^15^. Previous studies have demonstrated that HTLV-1-infected cells have an increased migratory capacity, which may facilitate the encounter with potential target cells^16^,^17^,^18^. We postulate that target cells are recruited to the vicinity of HTLV-1-infected cells through the effect of chemoattractants released by the latter^19^.

A recent study has reported that the levels of leukotriene B4 (LTB4) are higher in the plasma of HTLV-1-infected individuals than of uninfected individuals^20^. LTB4 is a potent chemoattractant that favours the recruitment of leukocytes to inflammation sites^21^,^22^,^23^,^24^,^25^. LTB4 is a metabolite of arachidonic acid (AA). It is synthesized by the sequential action of 5-lipoxygenase (5-LO) and leukotriene A4 hydrolase^26^. It mediates its effects by binding to the G-protein-coupled receptor BLT-1 (ref. 27). LTB4 is mostly secreted by neutrophils, mast cells, monocytes and macrophages^28^. Under physiological condition, lymphocytes do not secrete LTB4; the limiting factor for LTB4 secretion in lymphocytes is the synthesis of AA^29^,^30^,^31^,^32^. AA is released from membrane lipids by enzymes with a phospholipase A2 (PLA2) activity^33^. In the human genome, more than 30 enzymes possess a PLA2 activity; they are divided into nine separated groups, based on their structure, subcellular localization and enzymatic activity^34^.

In this study, we evaluate the role of LTB4 in HTLV-1 propagation. We demonstrate that HTLV-1-infected CD4 T cells secrete LTB4. We find that this secretion is the consequence of the overexpression of the cytosolic PLA2 gamma (cPLA2γ) induced by the viral transactivator Tax. We also show that LTB4 secretion facilitates the recruitment of T cells and virus transmission *in vitro*. Finally, we demonstrate that humanized mice infected with HTLV-1 and treated with a LTB4-inhibitor display fewer independent HTLV-1-infected cellular clones and lower HTLV-1 PVLs.

## Results

### Infected primary CD4 T cells spontaneously secrete LTB4

**Figure 1:**
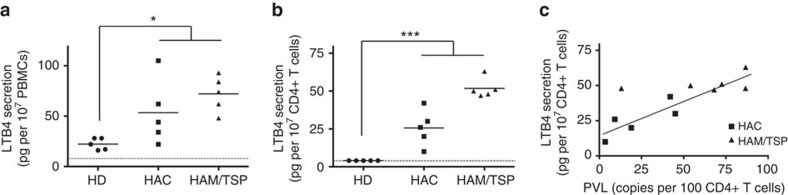
Primary CD4 T cells from HTLV-1-infected individuals secrete LTB4. (**a**) PBMCs from HTLV-1-infected individuals secrete significantly more LTB4 than PBMCs from HDs. PBMCs were culture for 30 min and spontaneous LTB4 secretion levels were determined by ELISA. Dashed line represents the detection limit of the ELISA kit. PBMCs were from HDs (*n*=5), HACs (*n*=5) or individuals who had developed HAM/TSP (*n*=5). Bars represent mean. *P* value=0.03; Mann–Whitney *U*-test (HDs versus HACs and HAM/TSP). (**b**) CD4 T cells from HTLV-1-infected individuals secrete detectable levels of LTB4. CD4 T cells were isolated, cultured for 30 min and spontaneous LTB4 secretion was detected by ELISA. Bars represent mean. *P* value<0.001; Mann–Whitney *U*-test (HDs versus HACs and HAM/TSP). (**c**) LTB4 spontaneous secretion by CD4 T cells correlates with the percentage of cells infected with HTLV-1. Pearson’s correlation test: *r*=0.87; *R*^2^=0.76; *P* value <10^−4^.

It was previously reported that, on ionomycin stimulation, PBMCs isolated from HTLV-1-infected individuals secrete significantly more LTB4 than PBMCs from uninfected healthy donors (HDs)^20^. Similarly, we observed that, in the absence of external stimuli, PBMCs from HTLV-1 asymptomatic carriers (HACs) or HAM/TSP patients spontaneously secrete higher levels of LTB4 than PBMCs from HDs (Fig. 1a).

We wondered if infected CD4 T cells could participate in such a secretion. Thus, CD4 T cells were isolated, and the LTB4 secretion capacity was assessed. As described before^35^, CD4 T cells isolated from HDs do not secrete detectable levels of LTB4 (Fig. 1b). In contrast, significant levels of LTB4 were detected in the supernatant of CD4 T cells isolated from HACs or HAM/TSP patients (Fig. 1b). Considering the proportion of CD4 T cells among PBMCs for the different donors, we estimated that 20–30% of the LTB4 spontaneously released by PBMCs is produced by CD4 T cells in HACs; in HAM/TSP, secretion by CD4 T cells accounts for 80% of the LTB4 released by PBMCs. Furthermore, LTB4 secretion levels positively correlated with the percentage of CD4 T cells infected with HTLV-1 (Fig. 1c).

These results on primary cells suggest that, in contrast to normal CD4 T cells, HTLV-1-infected CD4 T cells spontaneously secrete LTB4.

### LTB4 secretion depends on cPLA2γ overexpression by Tax

**Figure 2:**
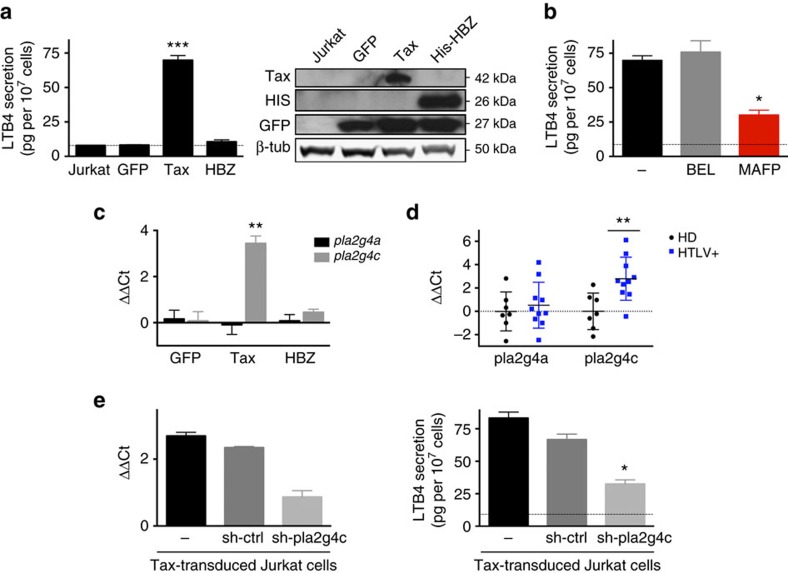
HTLV-1-induced LTB4 secretion depends on Tax-induced cPLA2γ overexpression. (**a**) Tax-expressing Jurkat cells secrete significant levels of LTB4. Jurkat cells were transduced with lentiviral vectors encoding GFP alone or together with the viral regulatory proteins Tax and HBZ. Protein expression was detected by western blot 48 h post transduction (right panel). Spontaneous LTB4 secretion levels were determined by ELISA. *P* value <10^−3^; *n*=4; mean±s.e.m.; ANOVA, Tukey’s *post hoc* test. (**b**) Tax-induced LTB4 secretion is dependent on group 4 phospholipases A2 (PLA2G4). Tax-expressing Jurkat cells were treated with either 1 μM MAFP or 5 μM BEL for 30 min. Spontaneous LTB4 secretion was detected by ELISA. *P* value=0.019; *n*=4; mean±s.e.m.; ANOVA, Tukey’s *post hoc* test. (**c**) *Pla2g4c* transcript levels are increased in Tax-expressing Jurkat cells. Expression levels of *pla2g4a* (encoding cPLA2α) and *pla2g4c* (encoding cPLA2γ) were determined by RT–qPCR on Jurkat cells transduced with GFP, Tax or HBZ. mRNA levels were normalized to *gapdh* expression and gene expression in Jurkat cells. *P* value=2.10^−3^; *n*=3; mean±s.e.m.; ANOVA test, Tukey’s *post hoc* test. (**d**) *Pla2g4c* mRNA levels are higher on CD4 T cells from HTLV-1-infected individuals when compared to HDs. CD4 T cells from HDs (*n*=7), HACs (*n*=5) and HAM/TSP (*n*=5) were isolated by negative selection. RT–qPCR for *pla2g4a* and *pla2g4c* was performed. Values were normalized to *gapdh* expression and the mean value on CD4 T cells from HDs. *P* value=0.007; mean±s.e.m.; Mann–Whitney *U*-test. (**e**) *Pla2g4c* KD counters Tax-induced LTB4 secretion. Jurkat cells were transduced with vectors encoding a shRNA-targeting *pla2g4c* (or a control sh-ctrl). Cells were then transduced with Tax. KD efficiency was determined by RT–qPCR (normalized to *gapdh* expression and *pla2g4c* in Jurkat cells) (left panel). LTB4 secretion was detected by ELISA. *P* value=0.03; *n*=3; mean±s.e.m.; Friedman test, Dunn’s *post hoc* test.

We set to determine which viral factor is responsible for HTLV-1-induced LTB4 secretion. To this aim, Jurkat cells were transduced with lentivectors encoding either Tax (the viral transactivator), HBZ (the viral regulatory protein encoded by the antisens transcript) or the reporter protein GFP as a control. Expression of the different proteins was confirmed by western blot (Fig. 2a, right panel). LTB4 was not detected in the supernatant of cells expressing either HBZ or GFP alone. On the contrary, Tax expression was sufficient to induce LTB4 secretion in Jurkat cells (Fig. 2a, left panel).

As the limiting factor for LTB4 secretion in lymphocytes is the PLA2 activity, we sought to identify which PLA2 protein is overexpressed in Tax-expressing cells. Thus, we tested the impact of PLA2 inhibitors on LTB4 secretion (Fig. 2b). Treatment with BEL (bromoenol lactone), an inhibitor of the group 6 PLA2s (PLA2G6s), did not affect Tax-induced LTB4 secretion. In contrast, MAFP (methyl arachidonyl fluorophosphonate) treatment, which inhibits the PLA2 activity of PLA2G4s and PLA2G6s, efficiently reduced LTB4 secretion by Tax-transduced Jurkat cells. We concluded that Tax-induced LTB4 secretion is dependent on PLA2G4s, also called cytoplasmic PLA2s (cPLA2s).

As the PLA2G4 family is composed of six members^36^, transcript levels for the six isoforms were measured by reverse transcription followed by quantitative PCR (RT–qPCR). Both on primary CD4 T cells and Jurkat cells, transcripts were detected only for two genes: *pla2g4a* and *pla2g4c*, which encode the proteins cPLA2α and cPLA2γ, respectively. While *pla2g4a* mRNA levels were not affected by Tax expression, *pla2g4c* levels were increased in Tax-transduced Jurkat cells (Fig. 2c). Similarly, *pla2g4c* mRNA levels were significantly higher in CD4 T cells isolated from HTLV-1-infected donors (HACs or HAM/TSP patients), than those isolated from HDs (Fig. 2d).

Although *pla2g4c* transcript levels were elevated, cPLA2γ could not be detected (by western blot or FACS). To confirm that LTB4 secretion depends on the cPLA2γ enzymatic activity, Jurkat cells were transduced with shRNAs targeting *pla2g4c* before transduction with Tax-encoding lentivectors. Knockdown (KD) efficiency was confirmed by RT–qPCR (Fig. 2e, left panel). We found that downregulation of *pla2g4c* prevented Tax-induced secretion of LTB4 (Fig. 2e, right panel), confirming the implication of cPLA2γ.

Overall, these findings demonstrate that, on HTLV-1 infection and Tax expression, cPLA2γ is overexpressed, leading to LTB4 secretion.

### Tax activates *pla2g4c* transcription through NF-κB and CREB

**Figure 3:**
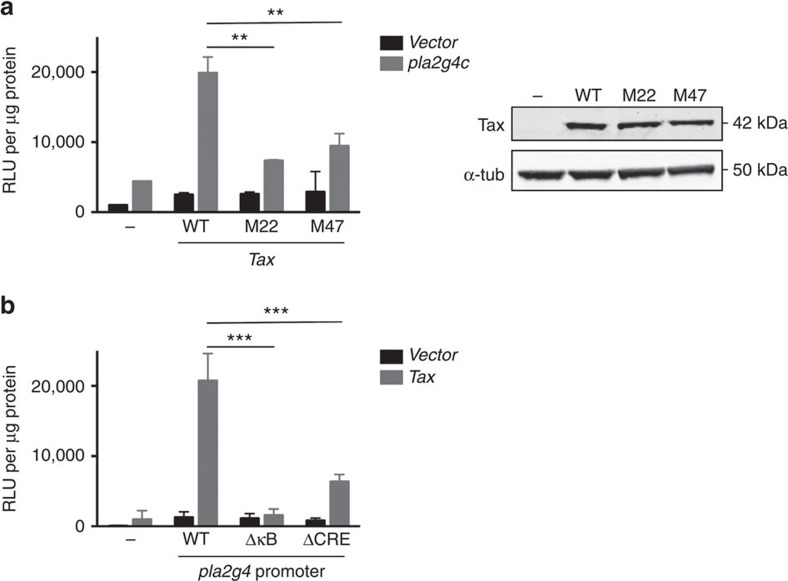
Tax-induced *pla2g4c* expression depends on both NF-κB and CREB pathways. (**a**) Tax mutants fail to activate *pla2g4c* promoter. *Pla2g4c* promoter was cloned into a luciferase reporter plasmid. 293T cells were transfected with the reporter plasmid (or the empty vector) and plasmids encoding Tax wild type (WT) or the mutants M22 (that do not activate the NF-κB pathway) or M47 (that do not activate the CREB pathway). Protein expression levels were detected by western blot 24 h post transfection (right panel). Luciferase activity (relative light units, RLU) was normalized to the protein concentration. *P* value=0.001; *n*=4; mean±s.e.m.; Two-way ANOVA, Sydak’s *post hoc* test. (**b**) The CRE and κB sites are both required for Tax-induced *pla2g4c* transactivation. 293T cells were transfected with a plasmid encoding Tax and with reporter plasmids with the luciferase gene downstream the WT *pla2g4c* promoter, or promoters depleted for the CRE or the κB sites (generated by site-directed mutagenesis). *P* value <10^−4^; *n*=4; mean±s.e.m.; Two-way ANOVA, Sydak’s *post hoc* test.

The *pla2g4c* promoter was cloned into a luciferase reporter plasmid. Transfection of the reporter plasmid together with a plasmid encoding Tax confirmed that the viral transactivator activates *pla2g4c* transcription (Fig. 3a).

Tax is known to activate multiple cellular pathways, including the CREB and NF-κB pathways. To determine which of these pathways are responsible for Tax induced *pla2g4c* expression, we tested the capacity of two Tax mutants (M22 and M47) to transactivate *pla2g4c*. These mutants are unable to activate the NF-κB and the CREB pathways, respectively^37^. Expression of the different mutants was confirmed by western blot (Fig. 3a, right panel). We found that both mutants failed to activate the *pla2g4c* promoter (Fig. 3a, left panel), suggesting that both CREB and NF-κB pathways are required for efficient Tax-induced *pla2g4c* transcription.

Previously, a κB site and a CRE site have been described on the *pla2g4c* promoter^38^. By directed mutagenesis of the corresponding sequences in the reporter plasmid, we observed that the promoter depleted for the κB site was no longer responsive to Tax and the one depleted for the CRE site was only partially inducible by Tax (Fig. 3b). These confirm the importance of both cellular pathways.

### Secreted LTB4 mediates potential target cell recruitment

**Figure 4:**
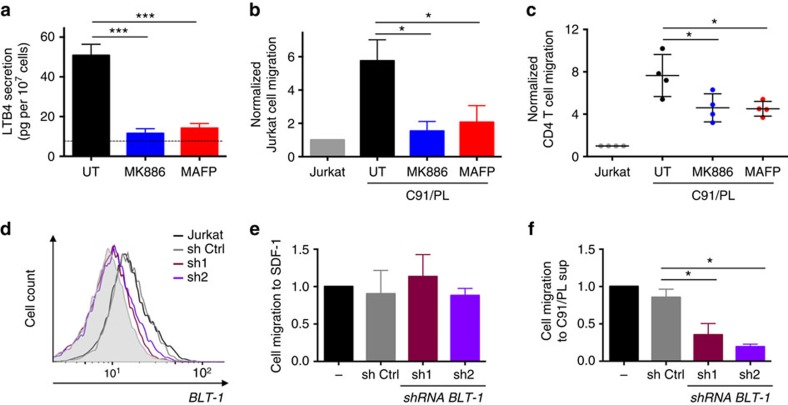
LTB4 secretion by HTLV-1-infected cells promote lymphocyte chemotaxis. (**a**) Drugs targeting the LTB4 synthesis pathway are efficient on C91/PL cells. C91/PL cells were pretreated with 200 nM MK886 or 1 μM MAFP (or left untreated, UT) for 30 min. Spontaneous secretion was determined by ELISA. *P* value <10^−4^; *n*=4; mean±s.e.m.; ANOVA, Tukey’s *post hoc* test. (**b**) The supernatant of C91/PL cells treated with either MK886 or MAFP fails at attracting Jurkat cells. The supernatant of pretreated C91/PL cells was added to the lower compartment of a Transwell device (5 μm of porosity). Jurkat cells (5 × 10^5^ cells cm^−1^) were added to the upper compartment. The number of cells that had migrated to the lower compartment was determined 1 h later. The number of cells migrating was normalized to the number of cells that had migrated towards the supernatant of Jurkat cells. *P* value=0.015; *n*=4; mean±s.e.m.; Friedman test, Dunn’s *post hoc* test. (**c**) Migration of CD4 T cells to C91/PL supernatant is reduced on either MK886 or MAFP treatments. The migration of primary CD4 T cells (10^6^ per cm) to culture supernatants in a Transwell device (3 μm of porosity) was determined as previously. *P* value=0.04; mean±s.e.m.; Friedman test, Dunn’s *post hoc* test. (**d**) BLT-1 expression was downregulated by transduction with lentivectors encoding shRNAs. BLT-1 expression on Jurkat cells transduced with shRNAs targeting *blt-1* (sh1 and sh2 in red and violet, respectively) or a control shRNA (in grey) was detected by flow cytometry. (**e**) *Blt-1* KD Jurkat cells migrate efficiently to SDF-1. The migration of the different Jurkat to SDF-1 (10 ng ml^−1^) was determined on a Transwell device. The number of cells that had migrated in 1 h to the lower compartment was then determined. Mean±s.e.m.; *n*=4. (**f**) Chemotaxis of Jurkat cells to C91/PL supernatant is reduced on *blt-1* KD. The different Jurkat cells were cultured in the upper compartment of a Transwell device, and C91/PL supernatant was added to the lower compartment. The number of cells that had migrated in 1 h to the lower compartment was then determined. *P* value=0.02; *n*=4; mean±s.e.m.; Friedman test, Dunn’s *post hoc* test.

To determine whether the LTB4 secreted by HTLV-1-infected cells favours the recruitment of potential target cells, we employed HTLV-1-chronically infected C91/PL cells, which secrete LTB4 at levels comparable to those secreted by primary infected CD4 T cells (Fig. 4a). LTB4 secretion can be inhibited using MK886, an inhibitor of the 5-LO cofactor FLAP, or MAFP (Fig. 4a).

The chemotactic potential of the C91/PL supernatant was analysed in a compartmentalized Transwell device. Jurkat cells (present in the upper compartment) migrated significantly more towards the supernatant of C91/PL cells than towards Jurkat cell supernatant (Fig. 4b). The supernatant of drug-treated C91/PL cells displayed a reduced chemotactic capacity on Jurkat (Fig. 4b), highlighting the importance of LTB4 in chemotaxis. Similarly, chemotaxis towards C91/PL supernatant was observed with primary CD4 T cells (Fig. 4c), and was partially inhibited on treatment of C91/PL cells with the different inhibitors (Fig. 4c).

We then generated Jurkat cells knocked down for the high-affinity LTB4 receptor BLT-1 (Fig. 4d). We verified that *blt-1* KD did not affect the overall migration capacity of the cells, as attested by the efficient migration towards stromal cell-derived factor 1 (SDF-1) (Fig. 4e). In contrast, *blt-1* KD Jurkat cells were deficient for chemotaxis to C91/PL supernatant (Fig. 4f).

These results emphasize the capacity of HTLV-1-infected cells to recruit potential target cells through LTB4 secretion.

### LTB4 favours conjugate formation and viral spread *in vitro*

To further evaluate the importance of LTB4 on HTLV-1 propagation, we determined the impact of blocking the LTB4 pathway on cell–cell contact formation and viral spread.

**Figure 5:**
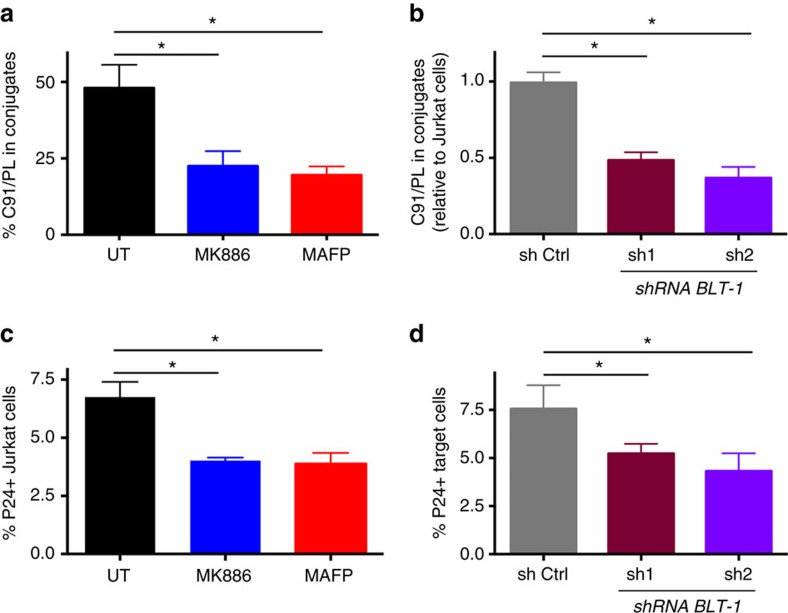
LTB4 secretion favours HTLV-1 viral transmission *in vitro.* (**a**) Blocking LTB4 production by C91/PL cells reduces the formation of cellular contacts with Jurkat cells. C91/PL cells were pretreated (or not) with either MK886 or MAFP. C91/PL and Jurkat cells were stained with different cell trackers to distinguish them. C91/PL and Jurkat cells were cocultured (ratio 1:1) for 30 min. The number of cell–cell contacts, or conjugates formed was then counted. *P* value=0.01; *n*=4; mean±s.e.m.; Friedman test, Dunn’s *post hoc* test. (**b**) *Blt-1* KD Jurkat cells form fewer contacts with C91/PL cells. C91/PL cells were cultured with *blt-1* KD Jurkat cells (sh1 or sh2), or Jurkat cells transduced with a control shRNA. The number of conjugates was then determined after 30 min. Data were normalized to the number of conjugates formed with Jurkat cells. *P* value=0.03; *n*=3; mean±s.e.m.; Friedman test, Dunn’s *post hoc* test. (**c**) Blocking LTB4 secretion by C91/PL reduces HTLV-1 viral transfer to Jurkat cells. C91/PL were treated or not with MK886 or MAFP, washed, stained with cell tracker and cultured for 1 h with Jurkat cells. The percentage of P24-gag-positive Jurkat cells was then determined by flow cytometry. *P* value=0.02; *n*=4; mean±s.e.m.; Friedman test, Dunn’s *post hoc* test. (**d**) *Blt-1* downregulation on Jurkat cells reduces HTLV-1 spread. C91/PL cells were stained with cell tracker, and cultured with *blt-1* KD Jurkat cells (sh1 or sh2), or Jurkat cells transduced with a control shRNA for 1 h. The percentage of P24-gag-positive Jurkat cells was then determined by flow cytometry. *P* value=0.04; *n*=4; mean±s.e.m.; Friedman test, Dunn’s *post hoc* test.

C91/PL cells were incubated with Jurkat cells for 30 min. Under the selected conditions (20,000 cells ml^−1^ per cell line), around 50% of the C91/PL cells were in contact with Jurkat cells, as quantified by double-blind counting on fluorescent microscope (Fig. 5a). On pretreatment of C91/PL cells with LTB4 synthesis inhibitors (MK886 or MAFP), the percentage of C91/PL cells contacting Jurkat cells was significantly reduced (Fig. 5a). Similarly, C91/PL cells formed fewer contacts with *blt-1* KD Jurkat cells, than with control shRNA-expressing Jurkat cells (Fig. 5b). Together these data indicate that LTB4 secretion by HTLV-1-infected cells favours the formation of cell–cell contacts.

We then cocultured C91/PL cells with Jurkat cells for 1 h, and monitored viral transfer by determining the percentage of Gag p24-positive Jurkat cells by flow cytometry. We found that inhibition of LTB4 production significantly reduced viral transfer to Jurkat cells (Fig. 5c). Likewise, viral transfer was significantly reduced towards *blt-1* KD Jurkat cells, when compared to sh-control Jurkat cells (Fig. 5d). In conclusion, LTB4 secretion by HTLV-1-infected lymphocytes participates in viral spread *in vitro*.

### Treatment with MK886 reduces HTLV-1 PVL *in vivo*

We have demonstrated that LTB4 secretion by cells chronically infected with HTLV-1 increases T-cell recruitment, cellular contact formation and viral transfer *in vitro*. This prompted us to investigate whether blocking leukotrienes could prevent HTLV-1 propagation *in vivo,* in a humanized mouse model.

**Figure 6:**
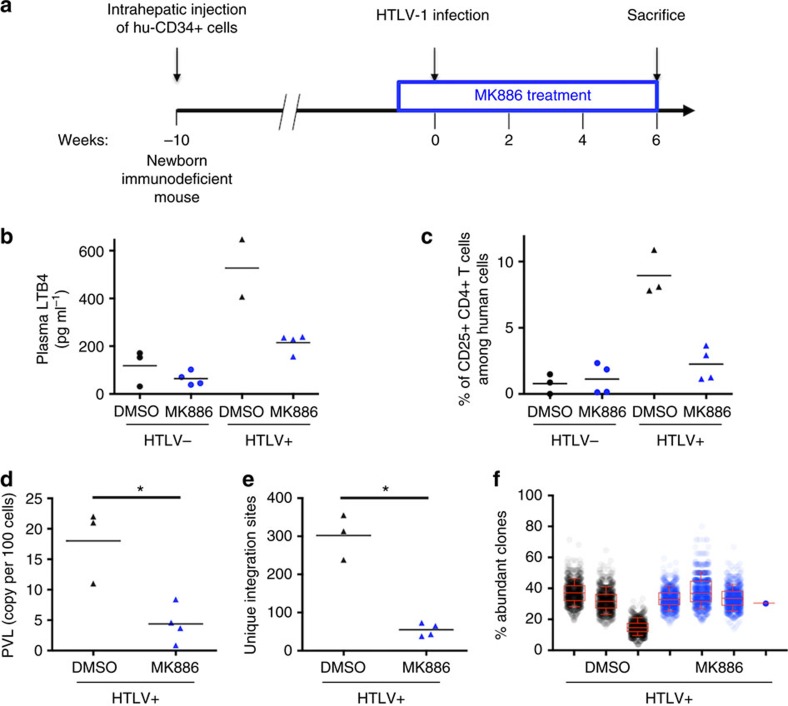
Blocking LTB4 synthesis during neoinfection *in vivo* results in lower HTLV-1 PVLs and a decrease in the number of infected clones. (**a**) Experimental procedure. Mice were considered humanized 8–10 weeks after CD34^+^ cell transplantation. Mice were then treated with IP injection of MK886 (5 nmol per mouse) three times a week. Injection with DMSO was used as control. One week after the initial injection, lethally irradiated MT2 cells were injected intraperitoneally. Six weeks after infection, mice were killed. Together, 14 mice were used. (**b**) LTB4 is increased in the plasma of HTLV-1-infected humanized mice. Six weeks after infection, plasma was collected and LTB4 levels were determined by ELISA. *P* value=0.006; bar represents mean. Kruskal–Wallis test. (**c**) The frequency of human splenocytes expressing CD25 is lower in infected animals treated with MK886. Six weeks after infection, mice were killed and splenocytes were collected. The percentage of human CD4 T cells (that is, hCD45^+^) positive for CD25 was determined by flow cytometry. *P* value=0.018; bar represents mean. Kruskal–Wallis test. (**d**) The PVL is lower in HTLV-1-infected mice treated with 5 nmol MK886. PVL is reported as the number of genomic *tax* copies per 100 human cells on splenocytes. *P* value=0.029; bar represents mean. Mann–Whitney *U*-test. (**e**) The number of UIS is lower in HTLV-1-infected mice treated with MK886. The number of independent HTLV-1-infected clones was determined by HTS clonality analysis (Supplementary Table 1). *P* value=0.028; bar represents mean. Mann–Whitney *U*-test. (**f**) The abundance of infected clones is not altered upon MK886 treatment. Abundant and non-abundant clones, defined by ≥2 or a single sequencing read, respectively, were determined for each animal by iterative subsampling (*N*=1,000) of an equivalent number of reads (*n*=59, Supplementary Table 1) within the total read number that supported LTR-host junctions. Dot plots represent the distribution of the percentage of abundant clones (number of abundant clones/total number of UIS) over 1,000 subsampling iterations for each animal. *P* value=0.48; Median, quartile and 10–90th percentile are presented in the Box-and-Whisker plot. Mann–Whitney *U*-test.

We generated humanized mice by inoculating immunodeficient mice with human CD34^+^ haematopoietic progenitor cells. Eight to ten weeks later, at a time when the human hematolymphoid system is well established, mice were treated with MK886 (or treated with DMSO as a control) and then infected with HTLV-1 by intraperitoneal (IP) inoculation of irradiated HTLV-1-infected MT2 cells (Fig. 6a). Mice were injected IP with either MK886 or DMSO thrice a week for 6 weeks after infection. Then mice were killed.

Consistent with a previous report in humans^20^, we found that LTB4 plasma levels were higher in HTLV-1-infected mice than in uninfected animals (Fig. 6b). Treatment with MK886 resulted in reduced LTB4 plasma concentrations (Fig. 6b).

We next determined the percentage of CD25^+^ CD4 T cells among hCD45^+^ splenocytes. Indeed, this percentage is a read out for high PVL in the mouse model^39^. As expected, HTLV-1-infected mice injected with DMSO showed an increase in the percentage of CD25^+^ human CD4 T cells compared to uninfected animals (treated either with MK886 or DMSO) (Fig. 6c). In contrast, the infected mice that were treated with MK886 showed a more modest increase, suggesting that the treatment may have affected HTLV-1 PVL. Consistent with these observations, we found that PVLs of MK886-treated mice were significantly reduced compared to those measured in DMSO-injected mice (Fig. 6d).

This decrease in PVL may either point to impaired clonal proliferation or a decrease in the number of HTLV-1-infected cell clones. To discriminate between these two hypotheses, we used an improved high-throughput sequencing (HTS) method to simultaneously map proviral integration sites and measure the abundance of the corresponding clones^40^.

The number of unique integration sites (UIS, corresponding to the number of independent HTLV-1-infected cellular clones) retrieved from MK886-treated mice was significantly lower than that from DMSO-injected animals, despite equivalent sequencing depths for both groups (Fig. 6e and Supplementary Table 1). Interestingly, increasing the sequencing depth for MK886-treated mouse samples did not alter the outcome regarding the number of retrieved UIS, suggesting that the system reached saturation.

We examined the patterns of clonal distribution and found that the relative abundance of HTLV-1-infected clones was not significantly different between MK886-treated and DMSO-injected mice (Supplementary Fig. 1). We next applied a more appropriate approach to compare clone abundance between groups of significantly different PVLs. This was achieved by comparing the abundance of HTLV-1-infected clones between MK886-treated and DMSO-injected mice by iterative subsampling, to correct for differences in the number of sequencing reads that support LTR-host junctions between samples, and thus for PVL. The proportion of abundant clones, defined by proviral integration sites supported by ≥2 reads, was equivalent between groups (Fig. 6f and Supplementary Table 2), strongly suggesting that MK886 treatment did not affect the potential of the HTLV-1-infected cells to expand. Consistent with this conclusion, using different thresholds (*n*≥3 and *n*≥4 reads per UIS) resulted in similar observations (Supplementary Table 2).

Altogether, our results demonstrate that MK886 treatment of HTLV-1-infected animals has an impact on the number of independent infected clones rather than on clonal expansion and cell proliferation. Our work underscores the critical involvement of leukotrienes in viral transmission and early phases of the HTLV-1 life cycle.

## Discussion

Unlike HIV-1, HTLV-1 infection occurs almost exclusively through cell–cell contact^41^. Infection purportedly occurs through viral synapse, transfer of viral biofilm or the formation of conduits^15^. While the mechanisms of viral transfer have been extensively described, little is known about the factors favouring target cell recruitment and contact formation. Here we show that HTLV-1-infected T cells are a source of LTB4, a potent chemoattractant, which participates in the recruitment of target cells and viral transmission, both *in vitro* and *in vivo*.

Trindade *et al*.^20^ have previously demonstrated that LTB4 levels are higher in the plasma of infected individuals than in the plasma of non-infected individuals. High plasma levels of LTB4 could be attributed to high secretion by neutrophils, which are potent LTB4 secretors and have an activated phenotype in HTLV-1-infected individuals^42^. In addition, Trindade *et al*.^20^ showed that PBMCs from infected individuals secrete larger amounts of LTB4 than control PBMCs, on ionomycin stimulation. They suggested that LTB4 is released by monocytes in response to viral sensing, since culture of PBMCs in the presence of viral particles was sufficient to induce LTB4 secretion, even in the absence of viral infection^20^. However, they have not considered the possibility of LTB4 secretion by lymphocytes, as normal T cells lack significant PLA2 activity and are incompetent for AA and LTB4 synthesis^35^. Strikingly, we demonstrate that primary HTLV-1-infected CD4 T cells spontaneously secrete LTB4 as the consequence of Tax-induced cPLA2γ expression. The increase in *pla2g4c* transcription on HTLV-1 infection was previously suggested by microarray and RNAseq studies^43^,^44^. Tax-induced *pla2g4c* transcription depends on both CREB and NF-κB activations. These two cellular pathways are also required in TNFα-induced *pla2g4c* induction in bronchoepithelial cells^38^.

The LTB4 level released by infected cells is sufficient to recruit lymphocytes (both from cell lines and primary CD4 T cells) *in vitro*. Although it seems to be a major factor, LTB4 may not be the only factor recruiting potential target cells. For example, HTLV-1-infected lymphocytes selectively recruit CCR4^+^ CD4 T cells *in vitro* via CCL22 secretion^45^. Authors suggested that CCL22 could be a major factor of target cell attraction, as infected cells express CCR4. However, a recent study demonstrated that CCR4 expression can be induced on infection and HBZ expression, hence it may not be initially expressed on target cells^46^. In contrast, BLT-1, the high-affinity LTB4 receptor, is expressed in a variety of inflammatory and immune cells, including macrophages, activated CD4 T cells, effector CD8 T cells and dendritic cells^27^. Thus, LTB4 could be involved in the recruitment of a larger set of target cells.

To examine the relevance of LTB4 for viral propagation *in vivo*, we employed a humanized mouse model of HTLV-1 infection^47^. We speculated that HTLV-1 propagation may be affected on inhibition of LTB4 production. We used MK886, a potent 5-LO inhibitor, which has been extensively used to block leukotriene secretion *in vivo*. We observed that MK886-treated mice displayed decreased PVL when compared to DMSO-treated HTLV-1-infected animals.

We verified by HTS mapping of HTLV-1 integration sites that this decrease was not dependent on altered proliferation of infected cells but rather on interference with virus propagation, consistent with the significant reduction in the number of independent clones observed on MK886-dependent inhibition of LTB4. Given the low PVLs observed in MK886-treated animals, we applied an improved HTS method, which includes several critical modifications in library preparation, and increases the sensitivity of the assay^40^.

While the oligoclonality index (OCI) introduced by Gillet *et al*.^48^ has been frequently used as a measure of clone abundance, OCI reflects the inequality of abundance between all clones of a given sample rather than their absolute abundance. In addition, OCI is highly dependent on sample size (in this case, the number of reads that support LTR-host junctions), with a particularly strong bias in the case of small samples^49^. Thus, to correct for the significant differences in PVLs observed between animals (low numbers of reads that support LTR-host junctions in MK886-treated mice), a subsampling method was applied to accurately compare clone abundances between groups. Using different thresholds for defining clone abundance, we showed that clone expansion *in vivo* is not affected by the perturbation of LTB4 secretion.

We have demonstrated that MK886 treatment affects HTLV-1 propagation during neoinfection. It is unclear whether blocking LTB4 has an impact on chronic infection: it has long been considered that HTLV-1 propagates in an individual mostly by clonal division of infected cells^50^. Thus, inhibiting LTB4 and viral propagation may have little impact. However, this dogma has been challenged by studies demonstrating the presence of markers of recent infection (two LTR episomal virus) both in primary infection and during persistent infection^51^,^52^,^53^. Moreover, the number of infected clones present in PBMCs isolated from HAM/TSP patients is significantly higher than in asymptomatic carriers^48^, suggesting that infection cycles during chronic infection may participate in HAM/TSP pathogenesis. Therefore, we postulate that targeting *de novo* infection may prevent PVL increase in asymptomatic carriers and HAM/TSP onset.

If the LTB4 pathway were to be considered as a therapeutic target, one should envision the inhibition of the upstream enzyme cPLA2γ. Indeed, with such an inhibitor, cPLA2α-dependent secretion, which is important in physiological and immune responses^54^,^55^,^56^, would remain unaffected. Moreover, cPLA2γ inhibition would also result in the reduction of other AA metabolites released by HTLV-1-infected cells, such as prostaglandins E2, which are involved in LTR activation and HTLV-1 viral expression^57^,^58^. Blocking cPLA2γ could reduce viral transmission both by preventing LTB4-mediated recruitment of the target cell, and reducing prostaglandins E2-mediated HTLV-1 replication.

In conclusion, this study demonstrates the critical role of LTB4 secretion in HTLV-1 transmission both *in vitro* and *in vivo*.

## Methods

### Cells

HTLV-1-chronically infected C91/PL and MT2 lymphocytes (Centre from AIDS reagents, NIBSC) and control Jurkat T cells (TIB-152, ATCC) were grown in RPMI (Gibco, Life Technologies) supplemented with 10% foetal bovine serum and 1% penicillin/streptomycin. HEK293T cells were grown in DMEM (Gibco) supplemented with 10% foetal bovine serum and 1% penicillin/streptomycin.

We obtained PBMCs from HDs, HTLV-1 asymptomatic donors and HAM/TSP patients in the context of a Biomedical Research Program approved by the Committee for the Protection of Persons, Ile-de-France II, Paris (2012-10-04 SC). All individuals gave informed consent. CD4 T cells were isolated from PBMCs by negative selection using magnetic beads (Miltenyi Biotech).

### LTB4 measurement and inhibitors

Cells were suspended at 10^6^ cells ml^−1^ in PBS and incubated for 30 min on ice. Cells were spun down at 400*g* for 5 min, resuspended (10^7^ cells ml^−1^) in RPMI (without serum) and incubated at 37 °C for 30 min. LTB4 secretion was stopped by addition of cold PBS, cells were centrifuged and supernatants were collected. LTB4 was then measured using an ELISA kit (Cayman Chemical).

When mentioned, cells were pretreated for 30 min with 200 nM MK886 (a FLAP irreversible inhibitor, Cayman Chemical), 1 μM MAFP (an inhibitor for both group 4 and 6 PLA2s, Cayman Chemical) or 5 μM BEL (an inhibitor for PLA2G6s, Cayman Chemical). Of note, these drugs are irreversible and washed away before testing.

### PVL quantification

CD4 T cells were isolated by positive selection and DNA extracted using the QIAamp DNA blood mini kit (Qiagen). HTLV-1 PVL was quantified through amplification and quantification of *tax* and *albumin* genes by TaqMan real-time PCR, as described previously^59^.

The primer set used to amplify HTLV-1 *tax* gene was F: 5′-CAAACCGTCAAGCACAGCTT-3′ and R: 5′-TCTCCAAACACGTAGACTGGGT-3′; the *tax* probe was 5′ 6-FAM-TTCCCAGGGTTTGGACAGAGTCTTCT-TAMRA-3′. The primer set for albumin was F: 5′-GCTGTCATCTCTTGTGGGCTGT-3′ and R: 5′-ACTCATGGGAGCTGCTGGTTC-3′ and the probe 5′-FAM-CCTGTCATGCCCACACAAATCTCTCC-TAMRA-3′. Runs were performed in a 20 μl volume containing 500 ng of total DNA extract, primers and probe (a 200 nM concentration of each), 1 × Maxima Probe/ROX qPCR Master Mix (Thermofisher). Thermocycling conditions were 2 min at 50 °C and 10 min at 95 °C, followed by 50 cycles at 95 °C for 15 s and 60 °C for 1 min. Quantification was standardized using DNA extracted from the HTLV-1-positive cell line MT4 (7 proviral copies per cell).

### Lentiviral vector production and transduction

Lentiviral vectors encoding GFP alone or together with Tax or His-HBZ were described previously^60^. Vectors were generated by transfection of HEK293T with psPAX-2 (encoding HIV Gag/Pol, 4.68 μg, Addgene), pMD2.G (VSV-G, 2.52 μg, Addgene) and pSD101-Tax/HBZ-IRES-GFP (9 μg) plasmids. Lentivectors encoding shRNAs were generated by transfection of HEK293T cells with psPAX-2, pMD2.G and pGIPZ plasmids encoding anti-*pla2g4c* shRNAs (V3LHS_368941), anti-*blt-1* shRNAs (V2LHS_112397 and V3LHS_336099 for sh1 and sh2, respectively) or non-targeting shRNA (CTRL sh) (GE Dharmacon). After 72 h, supernatants were collected, centrifuged, filtered at 0.45 μm and stored at −80 °C.

For protein encoding vectors, Jurkat cells were analysed 48 h post tranduction. Protein expression was determined by western blot (anti-Tax (1/1,000, Tab172, NIH), anti-GFP (1/8,000, clone JL-8, Clontech), anti-β-tubulin (1/2,000, D-10 polyclonal, Santa Cruz) and anti-His tag (1/1,000, clone H8, Abcam)). Uncropped scans of western blots are shown in Supplementary Fig. 2. For shRNAs, cells were selected for 10 days with puromycin (1 μg ml^−1^). KD efficiency was determined by RT–qPCR for *pla2g4c*, or by flow cytometry for BLT-1 (primary antibody: 30 μg ml^−1^, clone 202/7B1, Bio-Rad).

### Reverse transcription and quantitative PCR

Total cellular RNA was extracted using the RNeasy Plus Mini Kit (Quiagen). cDNA was synthesized from 500 ng of RNA using the superscript II reverse transcriptase (Invitrogen). mRNA levels of the different *pla2g4* genes were quantified by SYBR green-based qPCR using an Eppendorf realplex^2^ thermal cycler (15 min at 95 °C, 40 × (15 s at 95 °C, 20 s at 60 °C, 30 s at 72 °C)). GAPDH was used as a housekeeping gene. Primers were described previously^61^.

### Luciferase reporter assay

*Pla2g4c* promoter was amplified by PCR as previously described^38^, and cloned into a pGL2 basic luciferase vector (Promega). Site-directed mutagenesis was performed using the QuikChange II XL Site-Directed Mutagenesis kit (Agilent Technologies) and the primers previously used^38^.

HEK293T cells (6 × 10^5^) were transfected with pGL2-*pla2g4c* promoter plasmids and empty pSG5M or pSG5M-Tax (WT or mutants) plasmids using LipoD293 (SignaGen) following the manufacturer’s instructions. Luciferase activity was measured 24 h after transfection with the luciferase assay system (Promega) and chemiluminescence was detected using an EnSpire Multimode Plate Reader (PerkinElmer). The protein concentration was determined using the DC Protein Assay (Bio-Rad) to normalize for luciferase activity.

### Transwell assay

Jurkat migration to SDF-1 (10 ng ml^−1^, Sigma-Aldrich) or cell culture supernatant through a 5 μm-porosity Transwell filter (Corning) was determined by adding 2 × 10^5^ lymphocytes to the upper compartment and counting the cells present in the lower compartment after 1 h.

For the migration assay with primary cells, CD4 T cells were isolated from PBMCs by positive selection using magnetic beads (Miltenyi Biotech), cultured with IL-2 and activated with PHA for 24 h, and 5 × 10^5^ T cells were added onto a 3 μm-porosity Transwell device.

### Conjugate formation

HTLV-1-infected cells (C91/PL cells treated or not with the different drugs) and target cells (Jurkat cells transduced or not with shRNA encoding lentiviral vectors) were stained with distinct fluorescent dyes (CellTracker Red CMPTX or CellTracker Green CMFDA, 0.5 μM, Molecular Probes), and cocultured (ratio 1:1, that is, 20,000 of each cell type per ml) on 0.01% poly-L-lysine (Sigma-Aldrich) coated glass coverslips for 30 min at 37 °C. Cells were then fixed (2% paraformaldehyde) and mounted in DAPI Fluormount G (Southern Biotech). The percentage of C91/PL cells in contact with potential target cells was then determined by double-blind counting, by two distinct operators. For each replicate, at least 10 different fields were observed for each condition, corresponding to at least 300 counted HTLV-1-infected cells per condition.

### Analysis of cell-to-cell HTLV-1 transfer

HTLV-1-infected cells (C91/PL cells treated or not with the different drugs) were stained with a fluorescent dye (CellTracker Green CMFDA) and cultured (ratio 1:1, that is, 20,000 of each cell type per ml) with target cells (Jurkat cells transduced or not with shRNA encoding lentiviral vectors) for 1 h. Cells were then fixed with 2% paraformaldehyde, permeabilized with triton 0.05% and stained for intracellular p24 expression (Zeptometrix Corporation). Analysis was performed with a FACSCalibur flow cytometer (BD Biosciences).

### Isolation of human CD34^+^ cells from cord blood samples

Umbilical cord bloods were obtained from healthy full-term newborns with written parental informed consent according to the guidelines of the medical and ethical committees of Hospices Civils de Lyon and of Agence de la biomédecine, Paris, France.

After density gradient centrifugation of human cord blood, CD34^+^ cells were enriched twice using immunomagnetic beads according to the manufacturer’s instructions (CD34^+^ MicroBead Kit, Miltenyi Biotec). Purity (≥95%) was evaluated by FACS analysis using human PE-CD34 antibody (1/50, ref. 130-081-002, Miltenyi Biotec). Cells were frozen before the transplantation when newborn mice were available.

### Infection of humanized mice and sample analyses

NOD.Cg-Prkdc^scid^ Il2rg^tm1Wjl^ Tg(HLA.A2.1)1Enge/SzJ (NSG-HLA-A2/HDD) mice (Jackson Laboratory; males and females of 2–5 days of age) were bred and maintained under pathogen-free conditions. Newborn mice were sublethally irradiated with 1.1 Gray (320 kV, 25 mA; XRad-320, PXI Precision XRay) and injected intrahepatically with 2 × 10^5^ human CD34^+^ haematopoietic stem cells isolated from cord blood samples. After 8–10 weeks, humanized mice (≥30% CD45^+^ cells in peripheral blood) were first treated with MK886 (IP injection of 5 nmol per mouse) (or with the vehicle, DMSO) thrice a week. This treatment was maintained till the mouse was killed. One week after the initial MK886 injection, lethally irradiated MT2 cells (10^5^ cells per mouse) were intraperitoneally injected as previously described^39^. Mock-infected mice were injected with PBS.

Six weeks after infection, mice were killed. Blood was drawn on ACD and plasma collected on centrifugation and processed for ELISA. Spleens were collected and gently minced in PBS to obtain a single-cell suspension. Monoclonal antibodies provided by BD Biosciences were used for cell staining in a 1% BSA 0.1% sodium azide PBS buffer: Pacific Blue-hCD45 (1/250, ref. 560367), FITC-CD3 (1/50, ref. 555339), PE-hCD8 (1/50, ref. 555367), PE-Cy7-hCD4 (1/50, ref. 557852), APC-hCD25 (1/20, ref. 555434). Cells were incubated for 30 min in the dark at 4 °C with previously determined concentration of the relevant antibodies. Cells were gated to exclude doublets. Compensations were realized using Miltenyi MACS Comp Beads. Fluorescence was acquired using FACSCanto II and BDSDiva software (Becton Dickinson Immunocytometry Systems) and analysed using FlowJo software (Treestar). PVL was expressed as the number of copies of *tax* per 100 human cells as previously described^39^.

Animal experimentation was performed in strict accordance with the French ‘Comité National de Réflexion Ethique sur l’Expérimentation Animale, no. 15’ and the ethical guidelines for the care of these mice of the Plateau de Biologie Expérimentale de la Souris (PBES, UMS 3444) at École Normale Supérieure de Lyon. All efforts were made to minimize animal suffering.

### HTS clonality analysis

To determine the number and abundance of HTLV-1-infected clones in humanized mice, we used an improved quantitative HTS method to map the proviral integration sites in the human genome and simultaneously measure the abundance of the corresponding clones^40^. The method includes several critical modifications in library preparation and data analysis, overcoming some of the limitations of previously published protocols^48^. The dynamic range of the technique was increased by assaying both the 5′LTR and 3′LTR, allowing better determination of clone abundance. An extension step with Biotin-11-dUTP simultaneously end-repairs and facilitates streptavidin-based enrichment of LTR-positive fragments, increasing the sensitivity of the assay, followed by limited PCR to avoid PCR duplicates. Off-the-shelf Illumina primers replaced custom sequencing primers for the addition of adaptors and indexes, simplifying library multiplexing and reducing both the cost and hands-on time. Libraries were prepared starting from 500 ng DNA and sequenced on an Illumina MiSeq instrument. Hundred and fifty base pairs paired-end reads were acquired and sequencing reads that supported either the 5′ or the 3′LTR-host junctions were retained (Supplementary Table 1). The number of UIS and their abundance were determined.

To compare the abundance of the HTLV-1-infected clones between individuals and between groups, it was necessary to correct for differences in the number of sequencing reads that support LTR-host junctions between samples, and thus adjust for PVL. This was achieved by iterative (*N*=1,000) subsampling of an equivalent number of reads within the full data set of LTR-host reads obtained for each animal, the sampling size being determined by the animal with the lowest read number across all samples (*n*=59, filtered reads reported in Supplementary Table 1). For each animal, this yielded a number of UIS, a number of non-abundant clones (defined by the number of UIS supported by a single sequencing read) and a number of abundant clones (defined by the number of UIS supported by ≥2 reads) for each of the 1,000 subsampling iterations. Additional thresholds for defining clone abundance were also used (≥3 reads per UIS and ≥4 reads per UIS). Of note, for the sample with the lowest read number (59), each iteration consisted in the sampling of all reads. This generated *N*=1,000 times an identical value, symbolized by a single data point (Fig. 6f). Percentage abundant clones=number of abundant clones/total number of UIS.

### Statistical analyses

Analyses were performed using Prism software (v.6, Graphpad). Results were considered to be significant when *P*<0.05.

### Data availability

All relevant data are available from the authors on request.

## Additional information

**How to cite this article:** Percher, F. *et al*. HTLV-1-induced leukotriene B4 secretion by T cells promotes T cell recruitment and virus propagation. *Nat. Commun.* **8**, 15890 doi: 10.1038/ncomms15890 (2017).

**Publisher’s note:** Springer Nature remains neutral with regard to jurisdictional claims in published maps and institutional affiliations.

## Supplementary Material

Supplementary Information
