# Supplementary material for: HTLV-1-induced leukotriene B4 secretion by T cells promotes T cell recruitment and virus propagation
Source: Nat Commun. 2017 Jun 22;8:15890. doi: 10.1038/ncomms15890 (PMC5489682; doi:10.1038/ncomms15890)
Supplement: Supplementary Information [file ncomms15890-s1.pdf]

Title of file for HTML: Supplementary Information
Description: Supplementary Figures and Supplementary Tables

Title of file for HTML: Peer Review File
Description:

Supplementary Information

Treatment	Proviral load *(copies per 100 cells)*	Sequencing depth *(raw reads)*	Filtered reads *(LTR-host junctions)*	Number of UIS
**DMSO**	22	156905	4114	313
	11	136730	2508	238
	21	361365	2632	355
**MK886**	4.6	471666	155	65
	3.7	101706	284	38
	0.87	103966	59	43
	8.4	100406	245	73
P-value	***0.029***	*0.20*		***0.028***

Supplementary Table 1 – Blocking LTB4 synthesis *in vivo* results in reduced proviral loads and decreased numbers of independent HTLV-1 infected clones.

Proviral load was determined by qPCR. HTS of libraries prepared from DMSO- or MK886-treated mice resulted in similar numbers of raw read between the two groups. Reads corresponding to LTR-host junctions were retained and the number of UIS determined. P-values were determined by Mann-Whitney U tests.

Treatment	Number of UIS	Ab : ≥ 2 reads/UIS			Ab : ≥ 3 reads/UIS			Ab : ≥ 4 reads/UIS		
		Non-Ab	Ab	% Ab clones	Non-Ab	Ab	% Ab clones	Non-Ab	Ab	% Ab clones
DMSO	30	19	11	36.67	24	6	20	26	4	13.33
	28	19	9	32.14	23	5	17.86	24	4	14.29
	48	41	7	14.58	46	2	4.17	47	1	2.08
MK886	37	25	13	35.14	32	5	13.51	35	2	5.41
	12	8	4	33.33	8	4	33.33	9	3	25
	43	30	13	30.23	41	2	4.65	42	1	14.29
	28	19	9	32.14	22	6	21.43	24	4	2.33
P-value		***0.48***			***0.29***			***0.37***		

Supplementary Table 2 – MK886 treatment does not affect clonal expansion of HTLV-1 infected cells

Abundance of infected clones was examined for each animal by iterative sub-sampling within the set of reads that support LTR-host junctions (filtered reads, Supplementary Table 1). The sub-sampling size (n = 59 reads) was defined by the animal with the lowest number of filtered reads. Abundance was defined by the number of reads that supported each UIS (Methods). Clones were assigned to two classes of abundance (abundant clone: Ab, non-abundant clone: Non-Ab) based on three thresholds (2, 3 and 4 reads per UIS respectively). Median values of the number of UIS, the number of non-abundant and abundant clones (represented by read numbers below or ≥ thresholds of 2, 3 and 4 respectively), and the percentage of abundant clones determined over 1000 sub-sampling iterations are reported. P-value determined by Mann-Whitney U tests.

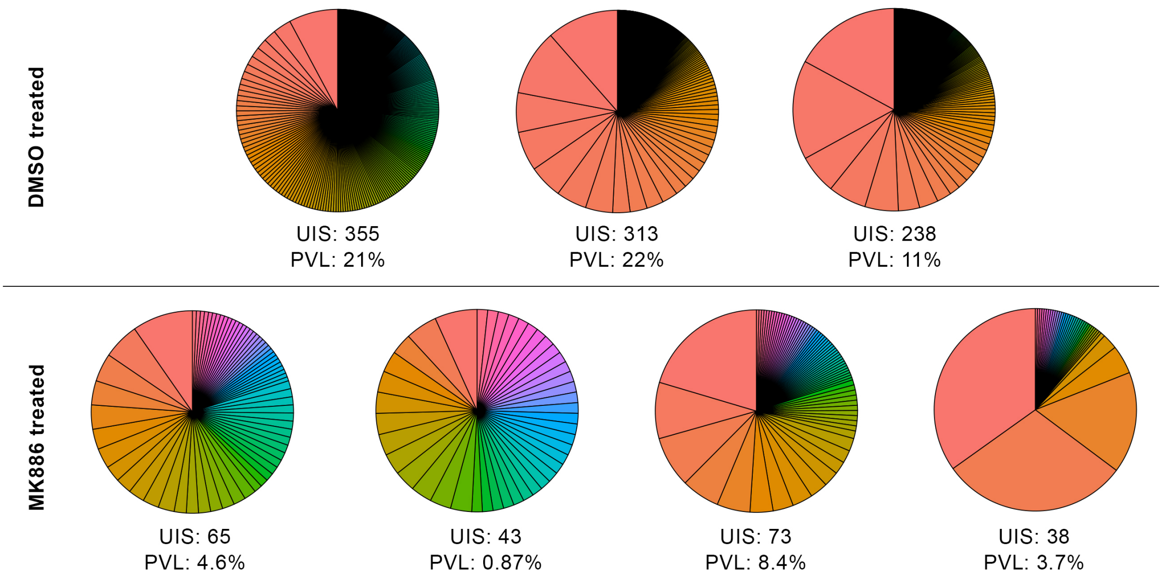

Supplementary Figure 1 - Relative abundance of HTLV-1 infected clones in MK886 treated and DMSO injected humanized mice

Clone frequency distribution of HTLV-1 infected cells in humanized mice is represented by pie-charts, each slice representing an independent integration site and its corresponding clonal abundance. UIS: number of unique integration sites. PVL: proviral load in PBMCs (*tax* copies per 100 cells). The oligoclonality index (OCI), a measure of clone abundance distribution, is not significantly altered in MK886 treated mice (p-value = 0.2, Mann-Whitney U test).

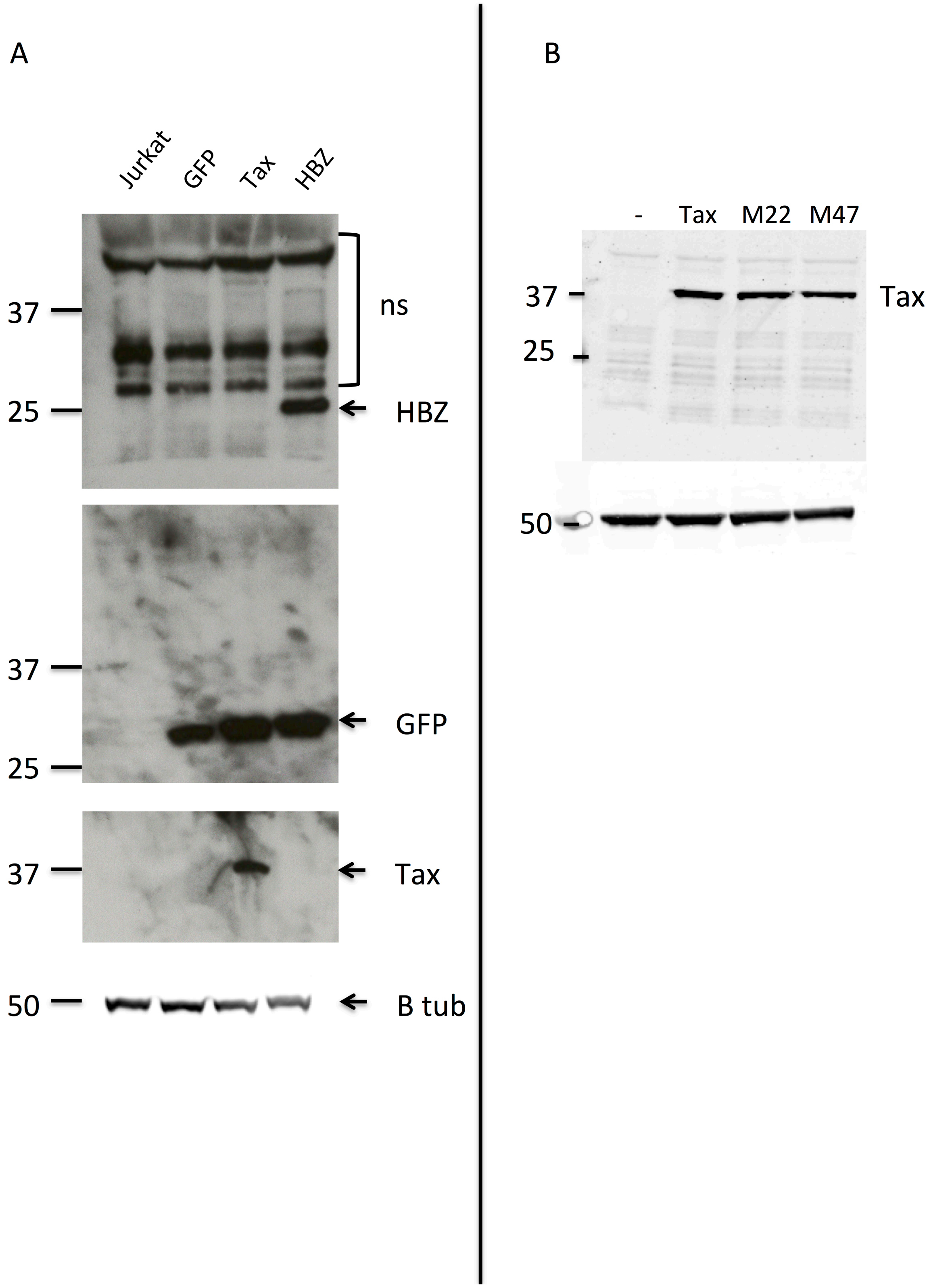

Supplementary Figure 2 – Original Western blots.

A- Figure 2; B- Figure 3
